# Primary Kaposi sarcoma of the subcutaneous tissue

**DOI:** 10.1186/1477-7819-6-94

**Published:** 2008-09-02

**Authors:** Liron Pantanowitz, John Mullen, Bruce J Dezube

**Affiliations:** 1 Department of Pathology, Baystate Medical Center, Tufts University School of Medicine, Springfield, MA, USA; 2 Department of Surgery, Beth Israel Deaconess Medical Center, Harvard Medical School, Boston, MA, USA; 3 Department of Medicine (Hematology-Oncology), Beth Israel Deaconess Medical Center, Harvard Medical School, Boston, MA, USA

## Abstract

**Background:**

Involvement of the subcutis by Kaposi sarcoma (KS) occurs primarily when cutaneous KS lesions evolve into deep penetrating nodular tumors. Primary KS of the subcutaneous tissue is an exceptional manifestation of this low-grade vascular neoplasm.

**Case presentation:**

We present a unique case of acquired immune deficiency syndrome (AIDS)-associated KS manifesting primarily in the subcutaneous tissue of the anterior thigh in a 43-year-old male, which occurred without overlying visible skin changes or concomitant KS disease elsewhere. Radiological imaging and tissue biopsy confirmed the diagnosis of KS.

**Conclusion:**

This is the first documented case of primary subcutaneous KS occurring in the setting of AIDS. The differential diagnosis of an isolated subcutaneous lesion in an human immunodeficiency virus (HIV)-infected individual is broad, and requires both imaging and a histopathological diagnosis to guide appropriate therapy.

## Background

Kaposi sarcoma (KS) is a low-grade vascular neoplasm associated with Human Herpesvirus-8 (HHV8) infection. There are four clinical-epidemiological types, including African (endemic) KS, AIDS-associated (epidemic) KS, classic KS, and transplant-associated (iatrogenic) KS. KS is a multifocal tumor that presents chiefly in mucocutaneous sites. AIDS-associated KS tends to be multicentric, often involving mucous membranes along the entire gastrointestinal tract and occurring in atypical locations. Patients with AIDS frequently manifest with skin lesions of the lower extremities, face, trunk, genitalia. In patients with AIDS, KS may also involve their lymph nodes and visceral organs. For patients with classic and transplant-associated KS, lesions are often limited to the skin, although visceral KS may occur. In African KS the legs are primarily involved, with more widespread KS involvement of the lymphoid system seen in children. Involvement of several unusual anatomical sites have been reported, such as KS of the musculoskeletal system, nervous system, heart, breast, major salivary glands, and endocrine organs [1].

Involvement of the subcutaneous tissue (subcutis or hypodermis) by KS typically occurs when cutaneous KS lesions evolve from a plaque stage lesion into deep endophytic nodular tumors. Large KS tumors may even penetrate deep down to involve underlying contiguous bone [2]. Hence, KS of the subcutis is, by and large, almost always accompanied by concomitant noticeable skin changes. We are aware of only one published case of AIDS-related KS involving the subcutaneous tissue of the thigh, that was associated with distant visible KS skin lesions of the patient's lower legs [3]. To the best of our knowledge, primary KS of the subcutis (i.e. without KS disease elsewhere) has not been documented. We present the first case of AIDS-associated KS primary to the subcutaneous tissue, in order to bring attention to the occurrence of KS in this unusual anatomical location.

## Case presentation

A 43-year-old homosexual man who was HIV positive for 18 years presented with a one-year history of a slowly enlarging mass in the proximal left anterior thigh. He described stabbing pain, often experiencing sharp shooting pains down the left thigh. He had been on and off antiretroviral medication, which he had stopped three years prior to this presentation. He had bilateral total hip replacements for avascular necrosis and osteoarthritis approximately three years prior to this visit. He reported no specific trauma or previous injection to his left thigh.

On physical examination, he appeared to be in good health. His gait was antalgic. He had no visible mucocutaneous KS lesions and he did not exhibit features of fat maldistribution. There was a firm 3 cm mass present deep in his left thigh that was tender to palpation. The mass was well away from the groin and inguinal region. In particular, there were no overlying skin changes or associated lymphedema. He had enlarged axillary lymph nodes. His complete blood count was unremarkable and his CD4 T-cell count was 249 cells/mm^3 ^and HIV viral load 72 copies/mL while off all antiretroviral medications.

**Figure 1:**
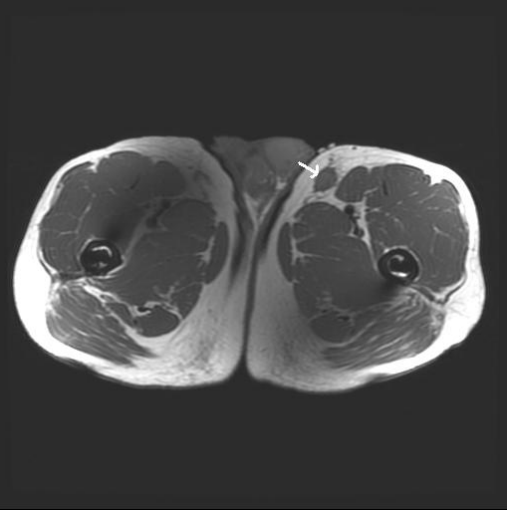
**MRI shows a solid, vascular enhancing subcutaneous thigh mass with spiculated margins.**(see arrow)

**Figure 2:**
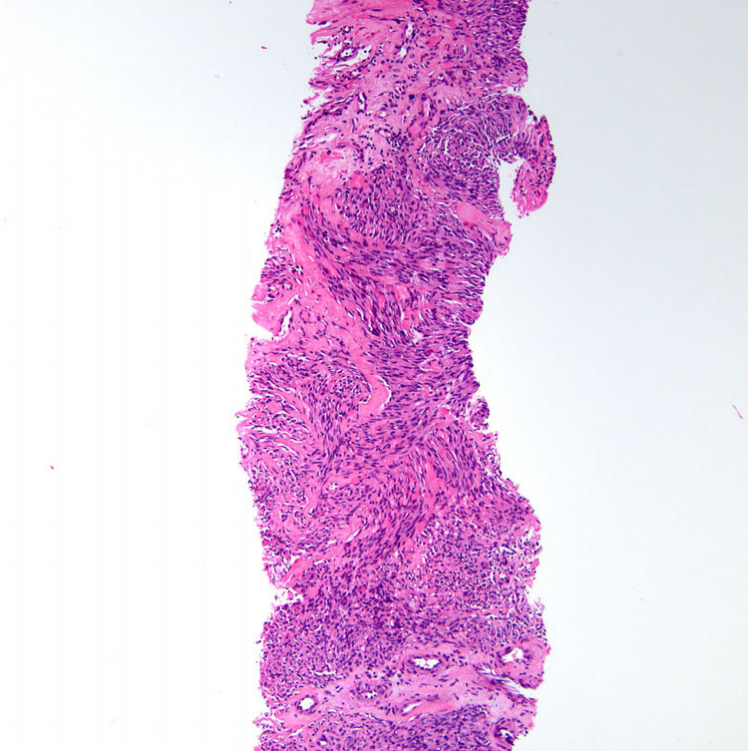
**Core needle biopsy of Kaposi sarcoma.** Fascicles comprised of spindled tumor cells are shown (H&E stain).

An ultrasound test showed a 2.6 × 1.8 × 1.2 cm solid, vascular, heterogeneous lesion within the deep thigh soft tissue. A magnetic resonance image (MRI) showed a solid, vascular enhancing mass with spiculated margins (Figure 1) located within the subcutaneous fat, superficial to muscle, in the left anterior thigh. The mass measured 2.2 cm in greatest diameter, and was associated with a second inferior satellite 1.4 cm subcutaneous tumor. Tumor was isointense to muscle on T1W1 and heterogeneous, but mostly hyperintense on T2WI. After gadolinium administration, both lesions enhanced. The larger index lesion enhanced heterogeneously and vessels were identified entering the proximal and distal aspects (Figure 2). No nodal disease was reported. Fecal occult blood test performed for evidence of gastrointestinal KS was negative and a chest x-ray showed no evidence of pulmonary KS.

**Figure 3:**
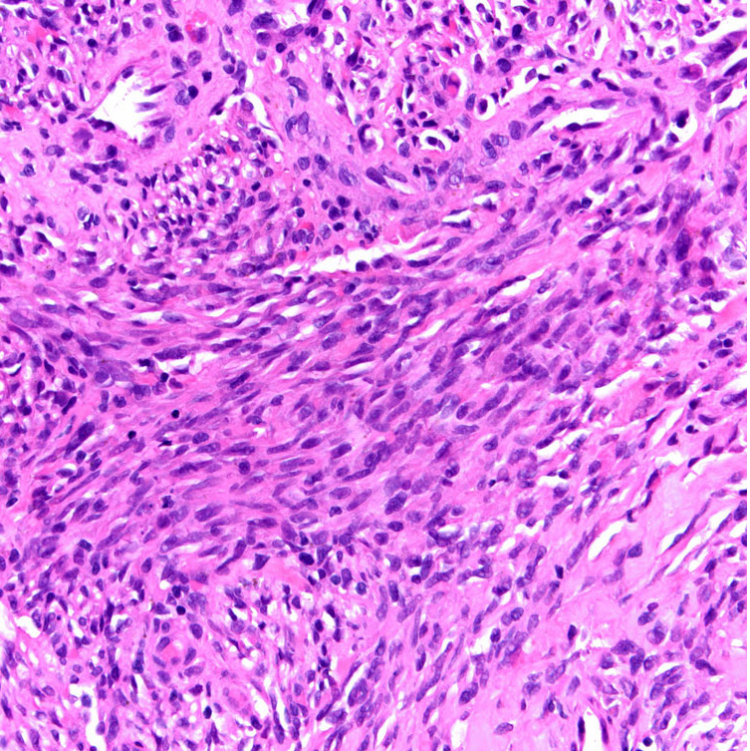
Higher power magnification shows infiltrating Kaposi sarcoma comprised of spindle-shaped tumor cells admixed with abnormal vascular channels (H&E stain).

Fine needle aspiration with a 22-gauge needle yielded only few atypical spindle cells. Therefore, an ultrasound-guided core biopsy was performed which showed KS with spindled tumor cells (Figure 3). KS tumor cells were immunoreactive for the vascular markers CD34 and CD31, for the lymphatic endothelial marker D2-40, positive for the HHV8 marker LNA-1, and demonstrated no staining with actin, desmin, cytokeratin cocktail, epithelial membrane antigen and S-100. The patient received pegylated liposomal doxorubicin with subsequent shrinkage of tumor and amelioration of his symptoms.

## Discussion

This report represents the first documented case of isolated KS manifesting primarily in the soft tissue of the thigh. Lee et al reported a case describing a subcutaneous AIDS-KS tumor in a 58-year-old HIV seropositive man that presented initially with KS skin nodules over his lower legs [3]. We are aware of another case of AIDS-KS in which the patient, a 57-year-old man, manifested with several subcutaneous noduli spread out over his entire legs [4]. This patient, however, presented with pronounced non-pitting lower extremity edema and visible KS skin plaques and nodules. In our case, there were no cutaneous changes at all.

The differential diagnosis of a subcutaneous thigh mass in an HIV-positive person is broad and includes infection (e.g. abscess, cryptococcus), reactive/benign conditions (e.g. nodular fasciitis), benign neoplasms (e.g. lipoma), and malignant neoplasms (e.g. liposarcoma, metastasis). The anterior thigh compartment is an uncommon site for an enlarged lymph node to manifest. Although infection should always be excluded in the context of immunosuppression, other than tenderness to palpation there were no findings prior to biopsy in our case that were particularly indicative of infection. Soft tissue abscess due to mycobacterial infection has been noted in patients with AIDS [5]. In our patient there was no antecedent trauma which may have caused localized nodular fat necrosis or fasciitis. Multiple subcutaneous lipomas induced by antiretroviral drugs have been reported [6]. More recently, leiomyosarcoma due to Epstein-Barr Virus (EBV) infection has emerged as a malignant soft tissue tumor that may arise in setting of HIV infection [7,8]. Of interest, there has been one case report in which AIDS-KS infiltrated the gastrocnemius muscle [9]. In our case there was no apparent involvement of skeletal muscle.

KS lesions develop as a result of the following combination of factors: HHV8, altered immunity (immunosuppression), and an inflammatory/angiogenic milieu [10]. The etiology for KS arising primarily in the subcutaneous (i.e. fatty subcutis) tissue is puzzling. KS has been shown to be of lymphatic origin [11], and lymphatic vessels are certainly present in subcutaneous tissue. However, KS tumorigenesis typically arises from dermal (superficial more often than deep) lymphatics in the skin, and not the hypodermis as in this case. Chronic lymphedema has previously been reported in several patients to promote KS development probably due to a combination of collateral vessels, lympahngiogenesis and immune impairment [12]. However, our patient reported no leg and or foot swelling and clinically we found no lymphedema. Localization of KS to sites of previous iatrogenic trauma has been documented [13,14]. It is plausible that trauma to our patient's thigh, perhaps related to his previous hip replacement, predisposed to him KS in this location. However, he had bilateral hip replacements and the KS lesion identified in this case was unilateral. Moreover, some of these publications describe KS arising after surgery relatively soon (e.g. within 6 days) after the patient's trauma [14].

In our case, imaging studies revealed a solid vascular subcutaneous mass with features highly concerning for malignancy. In such a case a definitive tissue-based diagnosis is key to guiding appropriate KS therapy. KS needs to be high in the differential diagnosis in the setting of HIV infection, to avoid a major sarcoma surgical operation. KS disease was not identified elsewhere in or patient, confirming the unusual diagnosis of primary subcutaneous KS. For a soft tissue abscess MRI will show a well-demarcated fluid collection that is hypointense on T1-weighted images, hyperintense on T2-weighted images, surrounded by a low-signal-intensity pseudocapsule with all sequences, and will likely demonstrate peripheral rim enhancement after intravenous administration of gadolinium-based contrast material [15]. For computerized tomography (CT) scans and MRI, AIDS-related KS is characterized by relatively strong tumoral enhancement after contrast material administration, a finding that may suggest the diagnosis in the appropriate clinical setting (ie, typical skin lesions), even though this finding is considered nonspecific [16]. CT is also helpful in assessing the involvement of deep tissue planes as well as the extent of possible nodal disease. Earlier imaging modalities, such as scintigraphy with sequential thallium and gallium scanning, have also been used to evaluate KS. Gallium uptake is usually negative in KS but positive in infection and lymphoma, whereas thallium uptake is positive in KS and lymphoma [17]. Finally, once a diagnosis of KS is a reached, considered an AIDS-defining neoplasm in an HIV-positive individual, appropriate therapy is required including HAART and if indicated chemotherapy.

## Conclusion

We present the first documented case of primary subcutaneous KS occurring in the setting of AIDS. The differential diagnosis of an isolated subcutaneous soft tissue tumor in an HIV-infected individual is broad, and requires imaging evaluation and a definitive pathological diagnosis in order to guide appropriate therapy. Awareness that KS can occur as an isolated deep soft tissue mass may avoid potential misdiagnosis.

## Competing interests

The authors declare that they have no competing interests.

## Authors' contributions

BJD, LP, and JM were involved in conception and design, in the drafting of the manuscript. All authors have read the final manuscript and approve of its submission.
